# Planarized THz quantum cascade lasers for broadband coherent photonics

**DOI:** 10.1038/s41377-022-01058-2

**Published:** 2022-12-24

**Authors:** Urban Senica, Andres Forrer, Tudor Olariu, Paolo Micheletti, Sara Cibella, Guido Torrioli, Mattias Beck, Jérôme Faist, Giacomo Scalari

**Affiliations:** 1 Quantum Optoelectronics Group, Institute of Quantum Electronics, ETH Zürich, 8093 Zürich, Switzerland; 2 Istituto di Fotonica e Nanotecnologie, CNR, Via del Fosso del Cavaliere 100, 00133 Rome, Italy

**Keywords:** Quantum cascade lasers, Integrated optics, Frequency combs, Optoelectronic devices and components

## Abstract

Recently, there has been a growing interest in integrated THz photonics for various applications in communications, spectroscopy and sensing. We present a new integrated photonic platform based on active and passive elements integrated in a double-metal, high-confinement waveguide layout planarized with a low-loss polymer. An extended top metallization keeps waveguide losses low while improving dispersion, thermal and RF properties, as it enables to decouple the design of THz and microwave cavities. Free-running on-chip quantum cascade laser combs spanning 800 GHz, harmonic states with over 1.1 THz bandwidth and RF-injected broadband incoherent states spanning over nearly 1.6 THz are observed using a homogeneous quantum-cascade active core. With a strong external RF drive, actively mode-locked pulses as short as 4.4 ps can be produced, as measured by SWIFTS. We demonstrate as well passive waveguides with low insertion loss, enabling the tuning of the laser cavity boundary conditions and the co-integration of active and passive elements on the same THz photonic chip.

## Abstract (web summary)

We present a high-performance integrated platform for broadband active and passive coherent photonics based on planarized THz quantum cascade lasers, featuring low losses and improved dispersion, RF and thermal properties.

## Graphical abstract

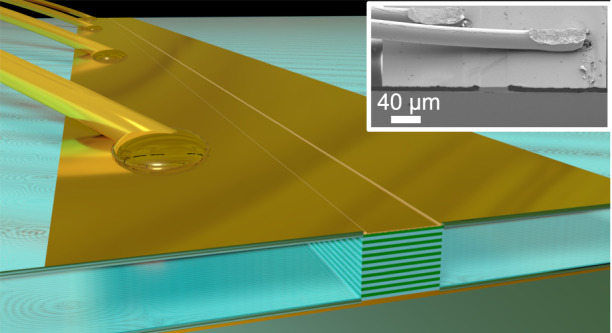

## Introduction

Integrated photonics^1^ makes extensive use of on-chip optical elements such as sources, splitters, modulators, and high-confinement waveguides embedded in a planar platform to efficiently process and route optical signals. There is a growing interest in integrated Mid-IR^2^ and THz photonics for telecommunications and sensing^3,4^. In the THz frequency range, a prominent candidate for source integration is the THz quantum cascade laser (QCL)^5^. Recent advances in the high-temperature operation of these devices^6,7^, combined with their frequency agility^8^ and the possibility to operate as frequency combs^9,10^ as well as very fast detectors^11^ make them extremely appealing as key building blocks for THz photonics. Some of the previous approaches to THz integration include hybrid plasmonic waveguides^12^, monolithically integrated THz transceivers^13^, coupled cavity devices^14^, and most recently devices integrated on silicon^15^.

In more complex photonic systems^16^, some of the crucial features for laser integration^17^ are the reduction of the electrical consumption, and consequently of the injected current, and the efficient coupling to low-loss passive waveguides. Here, we propose a new platform for integrated THz photonics that allows signal propagation with passive elements and coherent source integration for applications such as broadband sensing^18^ and coherent telecommunications^19^. In this first demonstration, we leverage the presence of a common metallic ground plane to demonstrate the integration of several active and passive THz photonic components onto the same semiconductor platform, allowing for efficient signal processing at THz and RF frequencies. We employ a low-loss polymer BCB, which has been used before for narrowband devices such as THz photonic crystal QCLs^20^ and antenna-coupled QCLs^21^. In our work, we focus on broadband and comb devices, highlighting improved performance in several crucial figures of merit such as dispersion, RF and thermal properties, and demonstrating the co-integration of active and passive elements on the same photonic chip.

## Results

### Planarized waveguide platform

**Fig. 1:**
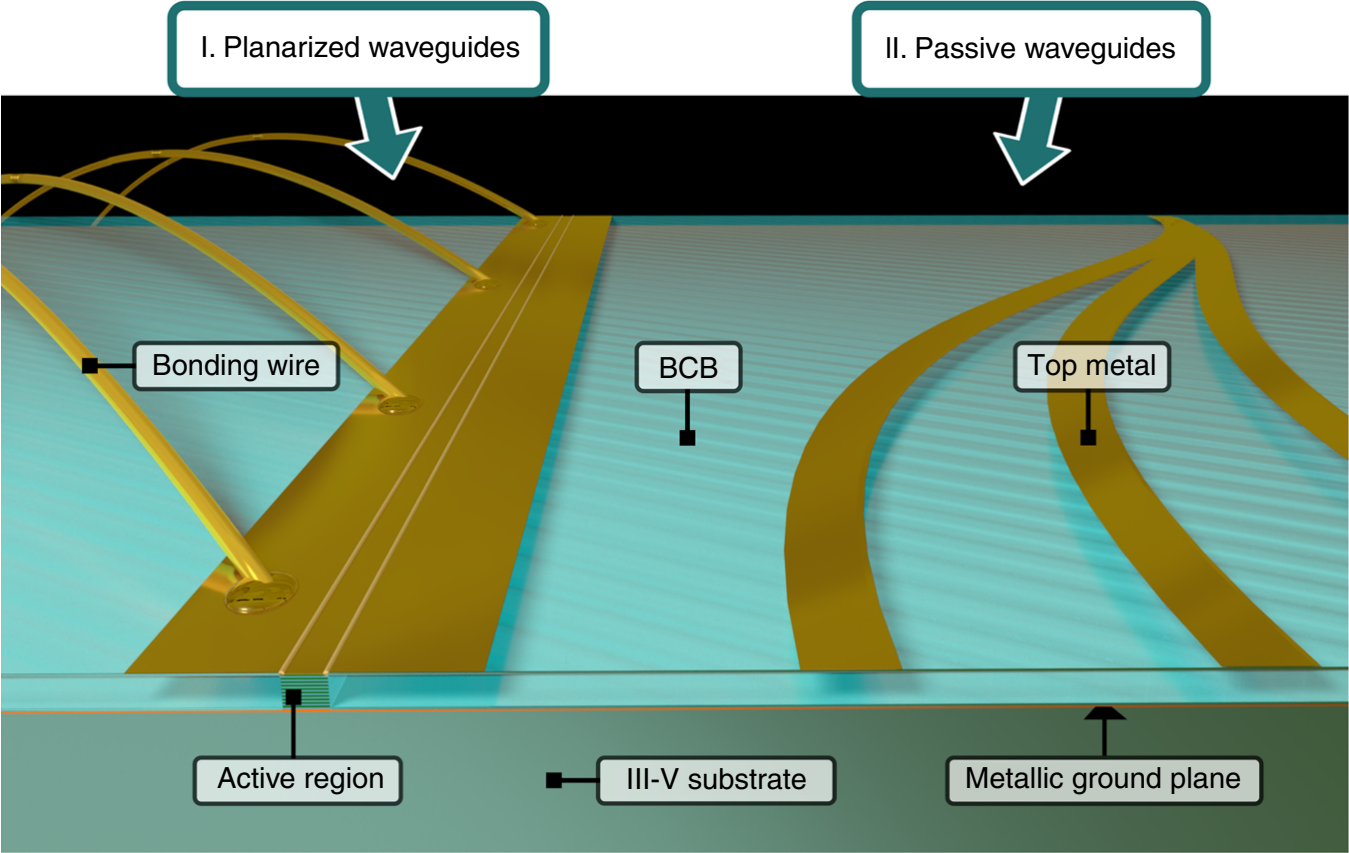
Our new platform for broadband coherent THz photonics is based on planarized active and passive waveguides embedded in BCB, a low-loss polymer. **I**. The planarized active waveguide consists of a standard double metal waveguide encompassed in BCB and an extended top contact metallization, which enables narrower active ridge geometries and the placement of bonding wires over the passive section. This configuration preserves the low waveguide losses of double metal waveguides while improving the dispersion, RF and thermal properties (see text for details). **II**. Passive waveguides can also be fabricated, consisting of metallic stripes on top of BCB, which provide confinement and guide the optical mode. These can be used to co-integrate active and passive elements on the same chip

The basic building block is a high-performance planarized double-metal waveguide with an extended top metallization, as shown in Fig. 1 on the left. A similar kind of waveguide has already proven to be very efficient both for THz and microwave applications^22^. Following a standard double metal waveguide fabrication process^23^ with dry-etched active region waveguides, a microelectronic-grade low-loss polymer benzocyclobutene (BCB) is spin-coated and baked as the surrounding material (see “Methods” for details). The latter is widely used in microelectronics and has already been successfully employed in several THz applications^21,24^. Specifically, we use Cyclotene 3022-57 (BCB), with a refractive index of 1.57 and relatively low losses (3 cm^−1^ = 1.3 dB/mm) at 3 THz^25^, making it an ideal planarization material. To ensure a flat and smooth profile, the BCB spin and bake step is repeated five times, and the top surface is subsequently etched with RIE to the same height as the active region waveguide. The RIE etching is performed in several steps, where intermediate investigation with a height profilometer and an optical microscope reveals the current height of the BCB layer and whether it has reached the height of the active region waveguides (visible interference fringes and color changes). No etching or redeposition of the exposed active region or metal occurs. Subsequently, an extended top metallization with a typical width of 300 µm is deposited over the active region and the BCB-covered area on the sides, which offers several advantages, as discussed below. From the active region standpoint, for these first demonstrations we used a strongly diagonal, low-threshold broadband GaAs/AlGaAs heterostructure, fully described in ref. ^26^.

We start by presenting results on the integration of on-chip THz frequency combs making use of planarized waveguides and optimized RF input and output coupling. In the context of THz frequency combs, we have shown that the control over transverse modes is essential in order to obtain a regular and flat-top comb spectrum^27^. The introduction of side absorbers mitigates lasing in higher-order transverse modes due to increased waveguide losses. A similar result can be achieved by reducing the transverse dimension of the laser ridge to 50 µm and below. However, for conventional double metal ridges, the width cannot be arbitrarily small since the waveguides are usually contacted by wire bonding directly on the top metallic cladding. This inherently limits the effective ridge width *w* to the dimensions of the bonding wire patch, making devices with ridges of 50 µm or below challenging to contact and prone to failures. Bonding directly on the active region can moreover introduce defects, increasing the waveguide losses and non-intentionally selecting specific modes, potentially compromising the long-term performance of the device and its spectral characteristics.

With our planarized platform, these issues can be solved. Placing the bonding wires on top of the extended top metallization over the passive, BCB-covered area, prevents the formation of any defects or local hotspots on top of the active region, and enables the fabrication of very narrow waveguides, well below the bonding wire size. The narrow waveguide width can be employed as an efficient selection mechanism for the fundamental transversal lasing mode and is also beneficial for heat dissipation and high-temperature continuous wave (CW) operation. With reduced waveguide widths we enter the regime of “wire” lasers^28^, which have a very favorable figure of merit for their surface-to-volume ratio as it scales as the inverse of the width S/V∝1/w, reducing the heating inside the active region waveguide. Moreover, the extended contact facilitates a lateral heat flow and eases the heat extraction as in a radiator scheme.

### Simulations

**Fig. 2:**
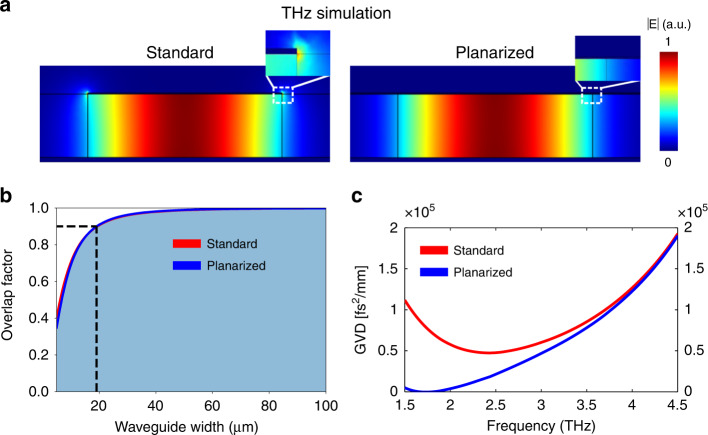
Numerical simulation results with a comparison of standard and planarized waveguide properties at typical THz QCL frequencies. **a** COMSOL 2D eigenmode simulation of a 40 μm wide waveguide at 3 THz. While there are field spikes present in the standard waveguide at the corners, for planarized waveguides the field distribution is smooth also at the environment boundary. **b** Computed overlap factors with the active region are comparable for the standard and planarized waveguides. The dashed line indicates the width where the overlap factor drops below 90%, for a frequency of 3 THz. **c** Computed chromatic dispersion for a waveguide width of 40 μm. At low frequencies (3 THz and below), the planarized waveguide has a significantly lower GVD than a standard waveguide

With the extended top metallization, the active region is sandwiched in a symmetric structure. As a consequence, the propagating optical mode does not feature any field spikes on the corners and at the edges of the ridge, as is the case for a standard double metal waveguide, as illustrated in Fig. 2a. One major advantage of using double metal waveguides is the large overlap factor of the propagating mode, reaching nearly unity. It can, however, reduce with the waveguide width. In Fig. 2b, we show that the computed overlap factor remains above 90% for ridge widths of above 20 µm for both the standard and planarized waveguides at a frequency of 3 THz. Since we are interested in frequency combs, another important figure of merit is the group velocity dispersion of the waveguide. In Fig. 2c we display results of COMSOL 2D eigenmode simulations, showing that the dispersion of the planarized waveguides is significantly reduced with respect to the standard ones, especially at low (*<*3 THz) frequencies. This is related to the change of the modal overlap factor as a function of frequency, as the evanescent field distribution changes differently for the standard and planarized waveguides. The computation includes waveguide and material (GaAs) dispersion for a 40 µm wide ridge waveguide. These 2D COMSOL eigenmode simulations can also be used to compute the waveguide (propagation) losses from the imaginary part of the mode effective index via the expression $\alpha = \frac{{4\pi k}}{{\lambda _0}}$, where *α* are the losses in units of cm^−1^, *k* the imaginary part of the mode effective index, and *λ*_0_ the wavelength in free space. For the same active waveguide widths, we obtain nearly identical propagation losses for the standard and the planarized waveguide (a difference of less than 1%). This is attributed to the large modal overlap factors, so the addition of a low-loss material (BCB) on the sides of the active waveguide does not have a significant contribution to waveguide losses, which are mainly caused by the overlap with the top and bottom metals. For example, for a 40 µm wide waveguide with a gold top and bottom metal, the computed waveguide losses at 3 THz are basically identical, 10.20 cm^−1^ and 10.13 cm^−1^ for the standard and planarized waveguides, respectively. In real fabricated devices, the sidewall roughness of the etched waveguides also plays an important role, resulting in increasing waveguide losses for narrower waveguide widths.

Heat dissipation properties are improved as well, as lateral heat transport takes place through the extended top metallization and the BCB polymer. COMSOL 2D thermal simulations show that for a 40 µm wide waveguide, the maximum temperature inside the active region is reduced by around 7 K in a planarized waveguide at maximum bias conditions (11 V, 400 mA/cm^2^) and a heat sink temperature of 100 K. A measurement study of the threshold current density as a function of increasing heat sink temperature^29^ and a comparison with data from our previous work^30^ also show a factor of 1.6 higher thermal conductivity of 130 W/Kcm^2^. More detailed results of these simulation and characterization studies can be found in the Supplementary Material.

The lateral heat transport could be eventually enhanced by functionalizing BCB with nanoparticles, as demonstrated in ref. ^31^, without affecting the waveguide’s optical losses. Moreover, as the planarized platform allows for the fabrication of even narrower waveguides, heating effects can be mitigated further. Recent results also display the positive impact of a thinner active region in obtaining high-temperature CW operation^32^. This approach can be as well combined with our planarized geometry to push the CW high-temperature operation even higher.

Next, we study the radio-frequency (RF) properties of the planarized waveguides. These are crucial both for the extraction and measurement of frequency comb beatnotes as well as for an efficient injection of RF signals which can affect and control laser operation. The relevant RF frequency range is close to the cavity repetition rate, defined as $f_{{{{\mathrm{rep}}}}} = \frac{c}{{2\;n_gL}}$, where *c* is the speed of light in vacuum, *n*_g_ the mode group index, and *L* the cavity length. For typical waveguide lengths between 2–4 mm, the corresponding *f*_rep_ lies roughly between 10 and 20 GHz, although harmonic comb states can generate RF beatnotes well above 50 GHz.

**Fig. 3:**
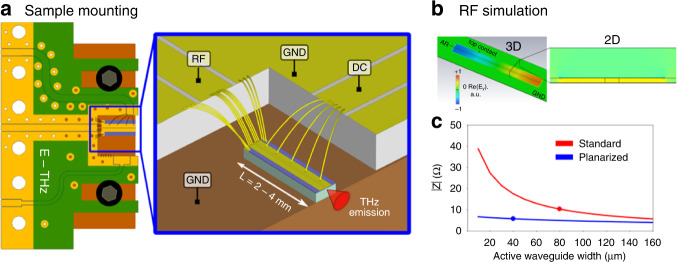
Optimized sample mounting and simulated RF properties of planarized waveguides. **a** Illustration of the sample mounting with a custom RF-optimized PCB. Several short signal wires are connected from a 50 Ω matched coplanar waveguide to one end of the waveguide, with short ground wires for minimal RF injection and readout losses (inset, top left). The DC laser bias is provided through a separate contact pad (inset, top right). **b** 2D and 3D eigenmode numerical simulations of the RF field at 20 GHz show that the whole extended top metallization encompasses a cavity with the ground plane. **c** Computed impedance from 2D COMSOL simulations for a frequency of 20 GHz and a varying active waveguide width. Typical waveguide widths are marked with circles

To fully capitalize on the improved RF properties, a dedicated RF PCB was developed and used for the laser mounting on copper submounts. As illustrated in Fig. 3a, the PCB features independent DC bias and RF readout/injection lines. The RF line is a straight 50 Ω matched coplanar waveguide which enables the placing of several short bonding wires at the backside of the laser waveguide, while short ground wires on each side close the loop to minimize RF losses. The positioning of all the RF signal wires on only one end of the waveguide is crucial in order to maximize the RF readout/injection efficiency, since the RF field produces a standing wave across the whole cavity with a node in the center and maxima at each end^33^, as shown in the simulation in Fig. 3b. A separate dedicated, non-matched contact pad provides the DC bias to the device, with bonding wires distributed along the whole length of the laser waveguide for a homogeneous current injection. The RF PCB is attached on the same copper submount as the laser chip with screws in such a position to minimize the distance between the RF port and the back facet of the laser, which is typically in the order of 0.5–1 mm.

2D and 3D eigenmode numerical simulations of the RF field of the planarized waveguide suggest that the whole extended top metallization encompasses a cavity with the ground plane, with the electromagnetic field oscillating across the whole patch, as shown in Fig. 3b for a frequency of 20 GHz. We also performed 3D numerical simulations of the whole relevant electromagnetic environment, including the bonding wires and the coplanar waveguide, as shown in the inset of Fig. 3a. Also these simulations result in the expected standing wave pattern in the planarized waveguide, suggesting that the bonding wires and the coplanar waveguides do not introduce any undesired standing wave or backshort effects between the RF PCB and the planarized waveguide. In addition, compared to standard waveguides, the RF properties of the planarized waveguides are modified in several aspects. First, due to the large modal overlap with BCB (low refractive index of 1.57), the effective index of the microwave mode $n_{{{{\mathrm{eff}}}}}^{{{{\mathrm{GHz}}}}}$ is reduced, which results in an increase of the fundamental microwave resonance frequency, detuning it from the THz mode spacing frequency *f*_rep_. Second, this also reduces the waveguide impedance at RF frequencies, as shown in Fig. 3c for a frequency of 20 GHz and a varying width of the active waveguide. Here, we performed 2D COMSOL simulations of the waveguide cross-section, where the refractive index of the active region was fixed to 3.6, while the metals were approximated as a perfect electric conductor (PEC). The lower impedance results in higher Q-factors of the microwave modes in the planarized waveguide, and in reduced radiative RF losses, due to a larger impedance mismatch to free space. Since the impedance of the planarized waveguide depends mostly on the width of the extended top contact, this allows for an independent design of the RF properties (e.g., by changing/modulating the width of the extended contact), while keeping the waveguide properties at THz frequencies unchanged. A more detailed analysis of RF properties with 3D numerical simulation results can be found in the Supplementary Material, which includes a more quantitative comparison between standard and planarized waveguides, supporting all the main conclusions presented here.

### Experimental results

**Fig. 4:**
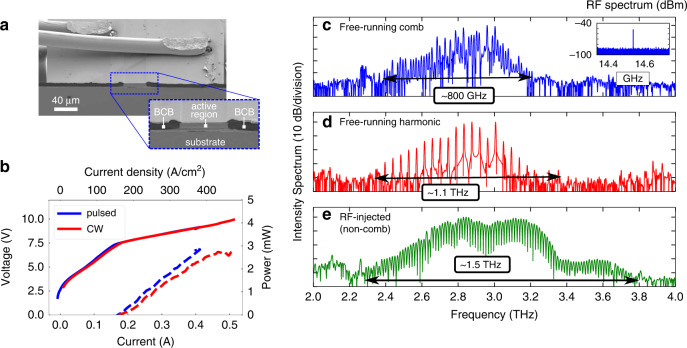
Measurement results of a planarized ridge waveguide device. **a** SEM image of a planarized, 40 μm wide ridge waveguide device, showing the cleaved front facet. The bonding wires are placed on the extended top metallization over the BCB-covered area. **b** LIV curves of a ridge laser sample measured in pulsed mode (500 ns pulses, 10% duty cycle) and in CW at a heatsink temperature of 40 K. **c** A free-running frequency comb spanning around 800 GHz, with the measured single strong RF beatnote (−55 dBm) shown in the inset. **d** A broadband free-running third harmonic state, covering a bandwidth of 1.1 THz. **e** With strong RF injection (+32 dBm at source) close to the free-running mode spacing, the emission spectrum can be broadened, spanning around 1.5 THz

We present now experimental measurements, first investigating the performance of simple ridge devices. These have a typical waveguide width of 40 µm, narrow enough for fundamental transversal mode selection, and wide enough for a large overlap factor and low propagation losses. Similar as in the case of standard double metal waveguides, a Fabry–Pérot cavity is formed by mechanical cleaving, which forms atomically flat end facets. In Fig. 4a we show an SEM image of the front part of the ridge waveguide with a cleaved facet and DC bonding wires on the extended top metallization. 3D electromagnetic numerical simulations show the formation of standing waves inside the cavity due to the finite end facet reflectivities (in this case in the order of 60% at 3 THz, very similar as for standard double metal waveguides).

Light-current-voltage (LIV) characteristics, shown in Fig. 4b, display a low-threshold current density in the order of 140 A/cm^2^ at a heat sink temperature of 40 K, which is due to the low- loss Cu–Cu planarized waveguide and the low-dissipation superdiagonal active region^26^. These planarized devices typically operate up to around 115 K in continuous wave (CW). A 40 µm wide and 2.7 mm long ridge waveguide with cleaved facets reaches output powers in the order of 3.0 mW at 40 K and 2.4 mW at 80 K in CW at rollover. The power was measured using a broad area absolute THz Power Meter by Thomas Keating Ltd (TK) in a lock-in measurement scheme (with micro/macro pulses or a mechanical chopper in pulsed and CW, respectively). The measured powers are from a single facet and uncorrected for any absorption in the cryostat window. Compared to standard double metal waveguides processed on the same layer, the threshold current density is higher than the best reference device from ref. ^30^ with a threshold current density of 110 A/cm^2^ at 40 K; however that device had a wider width of 65 µm and a longer length of 4 mm, resulting in lower waveguide and mirror losses. Moreover, for comb operation, additional lossy nickel side absorbers would be required, which would increase the waveguide losses. The peak output powers are comparable if we consider also the active waveguide dimensions, and the output efficiency is not expected to change, since the simulated end facet reflectivities are the same for both waveguides. A significant experimentally measured improvement is the maximum operating temperature of up to 115 K in CW, which is around 10 K higher than the best standard devices processed on the same epilayer as in refs. ^26,30^. This is consistent with the improved thermal properties (a more detailed and quantitative simulation and measurement comparison can be found in the Supplementary Material). We do not have a direct measurement comparison for a waveguide width of 40 µm, as this is not feasible for standard waveguides, while for planarized waveguides this is close to the optimal operation point considering waveguide losses, transversal mode selection, comb operation, and high-temperature CW performance.

In recent years, the possibility to obtain frequency combs from compact, on-chip sources^34^ has opened several possibilities from spectroscopy to LIDAR, remote sensing, and coherent communications^35^. Quantum-cascade lasers in the Mid-IR and THz proved to be excellent candidates for integrated, semiconductor-based comb sources^36^. We examine now the comb properties of our lasers based on planarized waveguides. Free-running devices can generate frequency comb states, where the THz modes are exactly equidistantly spaced, have a fixed phase relationship and produce a single RF signal (beatnote) at the mode spacing frequency^37^. A typical measurement result is shown in Fig. 4c, where the THz spectrum spans around 800 GHz and there is a single stable RF beatnote at the cavity repetition rate *f*_rep_. The measured free-running RF beatnotes in planarized waveguides can reach relatively high powers of −60 to −55 dBm at the spectrum analyzer readout, indicating improved RF properties (as described in more detail in the previous section). As a comparison, typical beatnote intensities for standard double metal waveguides with similar dimensions processed on the same epilayer usually reach values of around −70 dBm. Free-running beatnote maps of several devices can be found in the Supplementary Material. Moreover, self-starting pure harmonic states^38^^,^^39^ (Turing patterns) can be observed at specific bias points, where the mode spacing is an integer multiple of the fundamental *f*_rep_^40^. A typical harmonic state spectrum spanning over 1.1 THz is shown in Fig. 4d, in this case corresponding to the third harmonic.

By injecting an external RF signal to the laser cavity, it is possible to strongly modify the lasing operation both of a comb state^41,42^ or also of a high phase noise state^26,43^. In Fig. 4e we show an example where injecting a strong RF signal (+32 dBm at source) close to the natural cavity mode spacing (*f*_rep_ ± ≤200 MHz) can broaden the THz emission spectrum to over 1.5 THz. These are typically not frequency comb states due to the limiting chromatic dispersion over such a wide bandwidth (as evident already from the asymmetric interferograms in standard FTIR spectrum measurements^40^), but still very useful as sources of broadband THz radiation.

**Fig. 5:**
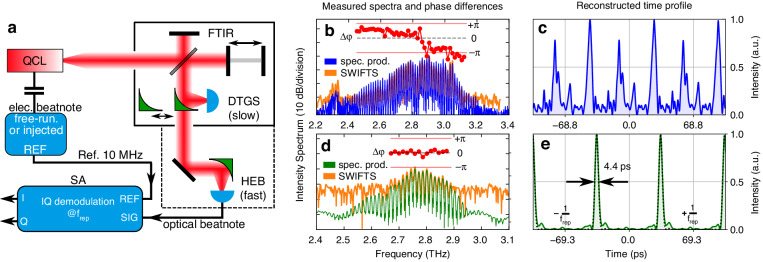
SWIFT Spectroscopy on a planarized ridge device with different RF injection powers. **a** Schematic of the SWIFTS setup, featuring an FTIR and a hot electron bolometer (HEB) as a fast detector. **b**, **c** Measurements of a weakly-injected free-running ridge device show relatively flat intermodal phase differences in two main groups, and the reconstructed time profile has a strongly amplitude-modulated periodic output intensity. **d**, **e** Strong RF injection (+32 dBm at the source) close to the repetition rate frequency on a ridge device close to lasing threshold results in active mode locking, producing pulses as short as 4.4 ps (close to the Fourier limit, dotted line). Here, the signal-to-noise ratio of the HEB measurement is reduced due to RF pickup problems with stronger RF injection

In order to assess the comb coherence and retrieve the phase between adjacent modes for reconstructing the time-domain emission profile, we used Shifted Wave Interference Fourier Transform Spectroscopy (SWIFTS)^9,44^. This is a coherent beatnote technique that requires a fast detector combined with an FTIR, as illustrated in the schematic in Fig. 5a. A hot electron bolometer (HEB)^45^ was used as the fast detector. It consists of a thin film (6 nm) NbN detecting element^46^, with a metallic (Ti/Au) log spiral antenna for an efficient in-coupling of the incoming THz radiation. Mounting and antenna coupling have been optimized to enhance RF performance. During operation, it is cooled down to the superconducting state (below 10 K) and features ultrafast rise times in the order of ∼40 ps^45,47^. When illuminated with a THz QCL frequency comb, the optical beatnote generated between adjacent THz modes can be measured directly on the bias line of the detector and fed into a spectrum analyzer with an IQ demodulator for SWIFTS^48^.

Here, we present two measurement examples in two different regimes. First, we analyze the comb state of a ridge waveguide device, injection-locked to the natural *f*_rep_ = 14.540 GHz with a relatively weak reference RF signal (+5 dBm at the source) in order to stabilize the repetition rate. The measured spectra and relative phases are shown in Fig. 5b, where the spectrum product measured with a slow DTGS detector (blue) and the SWIFT spectrum measured with the HEB (orange) have a good overlap and comparable signal-to-noise ratio, which is attributed to the high coherence of the comb state. Another factor is the different optical path that includes two more windows and an optical filter, reducing the total THz signal coupled to the HEB detector. The extracted intermodal phase differences are flat over a large part of the spectrum but separated into several groups. The reconstructed time profile produces a periodic waveform with significant amplitude modulation, in particular, several pulses on top of a background intensity, as shown in Fig. 5c.

The second example is the same ridge waveguide device operating just above lasing threshold and driven by a strong RF injection (+32 dBm at the source) at *f*_inj_ = 14.538 GHz, close to the natural repetition rate *f*_rep_ = 14.540 GHz. The measurement results in Fig. 5d show that we can reach an active mode locking regime^49,50^, where all the measurable modes have a flat relative phase profile, and the reconstructed time profile in Fig. 5e is a train of nearly Fourier-limited pulses as short as 4.4 ps. In this case, the SWIFT spectrum measurement with the HEB (orange) suffers from a worse signal-to-noise ratio as compared to the spectrum product obtained with the DTGS detector (green). This is attributed mainly to RF pickup problems due to the strong RF driving signal. Since only the central high-intensity modes could be measured with the HEB detector, the actual emitted THz pulses are very likely even shorter (assuming all the modes have the same phase).

We should also emphasize that for all the frequency comb results presented in this paper (including SWIFTS), the lasing modes throughout the full measured THz emission spectrum share both the same repetition frequency *f*_rep_ and the same offset frequency *f*_ceo_, and are thus part of the same comb. In the case of matching *f*_rep_, but several different values of *f*_ceo_, the spectrum consists of several (individual) sub-combs, in which case the SWIFTS time-domain reconstruction could not have been computed for the complete emission spectrum at once. Instead, it would be sliced into the contributions of each individual sub-comb. These aspects together with a thorough description of the FM to AM transition under RF injection are discussed elsewhere^48^.

### Passive waveguide components

**Fig. 6:**
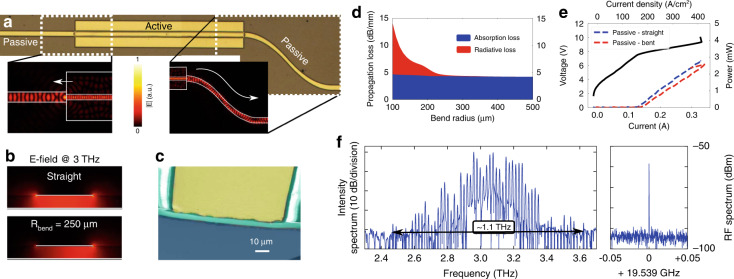
Simulation and measurement results of passive waveguides co-integrated with active planarized ridge devices. **a** Optical microscope image of a ridge device co-integrated with straight and curved passive waveguide elements, consisting of a metallized stripe on top of BCB (width 60 μm, mini-mum bending radius R = 500 μm). Insets show the simulated THz wave propagation with minimal scattering and bending losses at 3 THz. **b** Simulated electric field distribution in the passive waveguide cross-section at 3 THz. In the case of a straight waveguide, the intensity is concentrated symmetrically below the metal stripe, while it shifts to the outer side in the case of a bent path. **c** False color SEM images of the cleaved passive waveguide with a flat BCB end facet. **d** Computed passive waveguide propagation losses in dB/mm for a varying bend radius, obtained by COMSOL 2D eigenmode simulations at a frequency of 3 THz. The total losses are split into contributions from absorption loss (overlap with lossy metals and BCB) and radiative loss (bending loss), which is negligible for bend radii above 230 μm. **e** The LIVs measured in pulsed (500 ns pulses, 10% duty cycle, 20 K) show an increased threshold current density and a higher slope efficiency, both consistent with the lower end facet reflectivity. **f** Spectrum of a free-running device (CW, T = 20 K) in the comb regime with a bandwidth over 1 THz and a single RF beatnote above −60 dBm, indicating that the reduced cavity feedback can be beneficial for comb formation

Another important advantage of our planarized platform is the possibility to co-integrate active and passive elements. While we have already demonstrated planar reflective and outcoupling passive antenna structures^21,51^, here we designed passive waveguides for on-chip signal routing between various elements. Integration of passive waveguides is critical in the development of an integrated photonic platform. Near-infrared photonic circuits are nowadays a reality, and a similar approach can be envisioned for THz frequencies. An optical microscope image of a fabricated device is shown in Fig. 6a, where an active ridge is connected to straight and bent passive waveguides on each side. The passive waveguide is a metallized stripe on top of BCB which continues beyond the active region waveguide. The active waveguide has a width of 40 µm, while the passive waveguides have a slightly larger width of 60 µm to prevent any leakage to the sides due to possible lithographic misalignment. The minimum bending radius of the bent waveguide section is around R = 500 µm. Full-wave 3D numerical simulations show that the optical mode is guided below the metal stripe, following also non-straight paths (see insets in Fig. 6a). The reflectivity (coupling efficiency) at the active/passive waveguide interface can be tuned by the shape of the active and passive waveguides. In the simplest case of a flat active waveguide facet, a reflectivity of *R* = |*S*_11_|^2^ = 22.5% into the fundamental active waveguide mode, and a transmission of *T* = |*S*_21_|^2^ = 57.0% into the fundamental passive waveguide mode is obtained from a 3D numerical simulation at a frequency of 3 THz. This gives an insertion loss of $L_{{{{\mathrm{ins}}}}} = 1 - \frac{T}{{1 - R}} = 26.5\% = 1.34\;{{{\mathrm{dB}}}}$.

We performed 2D COMSOL simulations to evaluate the passive waveguide propagation losses. For a straight waveguide (field profile in the top panel of Fig. 6b), a propagation loss of around *α*_pass_ = 9.5 cm^−1^ = 4.1 dB/mm at 3 THz is obtained. This originates mainly from the non-optimized optical mode overlap with the top and bottom metal layers (a copper/gold stack), as BCB has a relatively low loss of 3 cm^−1^ = 1.3 dB/mm at around 3 THz^25^. If we consider a waveguide bend with a curvature radius *R*_bend_, the electric field intensity is shifted towards the outer part of the passive waveguide, as shown in the bottom panel in Fig. 6b. Below a certain critical radius *R*_bend_, which depends on the frequency and passive waveguide width, the propagation losses start to increase exponentially due to bending losses. In Fig. 6d, we plot the propagation losses of a passive waveguide with a top metallic stripe width of 60 µm at 3 THz. The radiative losses are negligible for a bend radius above around 230 µm, slightly above two times the free-space wavelength. This value however does not impose a definitive limitation for the integration density of passive components, as it is possible to implement, for example, low-loss sharp waveguide bends with inverse-designed optimized components^52^.

To characterize the passive waveguides experimentally, we performed a mechanical cleaving around 1 mm away from the active-passive waveguide interfaces on both sides and mounted the sample on a custom holder to measure the laser output from both sides of the device (straight and bent). The active region waveguide has a width of 40 µm and a length of 2 mm. Compared to cleaved active ridge facets, due to refractive index matching and the continued top metallized stripe, the end facet reflectivity reduces from around 60% to around 22.5%, as predicted by 3D numerical simulations. A false-color SEM image of the cleaved passive waveguide can be seen in Fig. 6c. The measured LIV curves in pulsed (500 ns pulses, 10% duty cycle, 20 K) are shown in Fig. 6e. The peak output powers from the straight (1 mm long) and bent side (around 1.2 mm long) of the device are comparable, indicating low propagation and bending losses. The threshold current density increased to around 160 A/cm^2^ due to higher mirror losses. Using the mirror loss formula $\alpha _{{{{\mathrm{mirr}}}}} = - \frac{1}{{2L}}\ln \left( {R_1R_2} \right)$ and comparing a 2 mm long waveguide with cleaved facets (*R*_1_ = *R*_2_ = 60%) versus coupling on both sides to a passive waveguide (*R*_1_ = *R*_2_ = 22.5%), the mirror losses increase from 2.6 cm^−1^ to 7.5 cm^−1^. The slope efficiency of the measured samples is increased from 11.9 mW/A to 13.6 mW/A. This is not as large as anticipated from the reduced facet reflectivity, however we need to take into account that the tested sample is coupled to a relatively long (around 1 mm) passive waveguide, which induces propagation losses. A simple back-propagation calculation gives us an upper estimate of the power emitted from the active facet (without absorption losses in the passive section): $P^\prime = P\;{\rm{e}}^{\left( {\alpha _{{\rm{pass}}}L} \right)} = 7.1\;{{{\mathrm{mW}}}}$. Here, we used the numerical simulation result of *α*_pass_ = 9.5 cm^−1^ and *L* = 1 mm. This would yield an increased slope efficiency of 37.6 mW/A. Although the actual measured power is lower, it could be reached with a shorter passive waveguide, and possibly increased further by using a passive outcoupling structure as in ref. ^21^.

The frequency comb performance does not deteriorate, but is actually improved. In Fig. 6f we showcase the largest measured comb bandwidth, broader than 1 THz, and a single RF beatnote stronger than −60 dBm. Here we should note that this sample was mounted without the custom RF PCB from Fig. 2d, but with simple metallized ceramic pads positioned close to the laser facet, which are estimated to increase the RF coupling losses in the order of 5–10 dB. As anticipated in the Mid-IR by numerical results in ref. ^53^ and theoretical models in ref. ^54^, the product of the facet reflectivities of a QCL ridge laser affects the maximum bandwidth obtainable by a given gain medium, and a lower reflectivity should enhance the comb bandwidth. In particular, the maximum bandwidth is achieved for a product $R_1R_2 \approx 0.2$. In our case, the lower reflectivity at the ends of the double metal cavity due to the coupling to the passive section brings the product $R_1R_2|_{{\rm{active}}} = 0.36\;{{{\mathrm{to}}}}\;R_1R_2|_{{\rm{act}} + {\rm{pass}}} = 0.062$.

Using different combinations of cleaved and passive waveguide facets, the planarized geometry offers the possibility to change the cavity reflectivity, adding an important element to the comb engineering toolbox. This could be expanded further by fabricating more complex dry-etched waveguide facet geometries, for example an adiabatic taper coupled to a passive waveguide, to make the reflectivity even lower. Finally, coupling broadband frequency combs to passive waveguides on the same chip, as already demonstrated in the Mid-IR^55^, is an important milestone towards fully integrated active and passive THz photonic circuits and spectrometers.

## Discussion

In conclusion, we have presented a novel platform for broadband coherent THz photonics based on high-confinement active and passive planarized double metal waveguides. The extended top contact metallization enables bonding wires to be placed over the BCB-covered area, which results in low waveguide losses, improved dispersion, RF and thermal dissipation properties, and allows for an independent design of THz and RF waveguide properties. The fabrication of narrow waveguides acts as a fundamental mode selection mechanism and further improves heat dissipation. Free-running broadband frequency combs over 800 GHz and harmonic states over 1.1 THz are observed. Driven with an additional external RF signal, broadband THz emission spectra over nearly 1.6 THz, and actively mode-locked pulses as short as 4.4 ps can be generated on demand. The co-integration of passive elements with custom on-chip guiding, reflection and outcoupling properties complete the diverse integrated THz photonics toolbox enabled by our planarized platform.

## Methods

### Planarized waveguide fabrication

From MBE-grown wafers, samples with a typical size of (9 × 10) mm^2^ were cleaved. A metallic stack of Ta(5 nm)/Cu(250 nm)/Ti(50 nm)/Au(500 nm) was deposited on the sample and on a carrier n+ GaAs substrate by electron beam evaporation. These were bonded using thermocompression wafer bonding at a temperature of 320 °C and a pressure of 5 MPa for 15 min in vacuum. After a mechanical thinning and several wet etch steps to expose the active region, the waveguides were dry-etched in a Cl_2_/H_2_ inductively-coupled plasma (ICP) using SiNx as a hardmask. Subsequently, AP3000 was spun on the sample as an adhesion layer for BCB (3000 RPM for 30 s). Cyclotene 3022-57 (BCB) was both spin coated (5000 RPM for 50 s) and baked sequentially five times (4x softbake at 210 °C and 1x hardbake at 250 °C for 2 h). This resulted in a total BCB thickness of around 25 µm to ensure a smooth profile across the sample. Using Reactive Ion Etching (RIE), the BCB was etched in several steps with intermediate inspection with a height profilometer and an optical microscope. The appearance of visible interference fringes and later a brighter color of the active waveguide surface marked the point when the BCB reached the height of the active region (10.4 µm). Finally, the extended top metallization (same metallic stack as the wafer-bonded side) with a typical width of 300 µm was defined with photolithography and lift-off, spanning over the active waveguides and the BCB-covered area on the sides. The passive waveguides were fabricated over BCB-covered areas in this final step as well.

### RF mounting

The custom two-layer RF PCB was fabricated on a Rogers 4350 substrate with a thickness of 0.8 mm. The coplanar waveguide has a width of 800 µm and is separated from the side ground planes by 100 µm. A series of vias connecting to the ground plane improves the RF performance and the isolation between RF and DC ports. The PCB is screwed to a copper laser submount and mounted on a cryostat cold finger. It is connected with a standard RF connector (HK-LR-SR2(12)) to low-loss semi-rigid RF cables (SUCOFORM 86 FEP), accessible from the outside of the cryostat.

### Supplementary information

Supplementary Material

## Data Availability

All the simulation and experimental data supporting this study are available from the corresponding author upon reasonable request.
