# Author Correction: Planarized THz quantum cascade lasers for broadband coherent photonics

**DOI:** 10.1038/s41377-024-01467-5

**Published:** 2024-07-04

**Authors:** Urban Senica, Andres Forrer, Tudor Olariu, Paolo Micheletti, Sara Cibella, Guido Torrioli, Mattias Beck, Jérôme Faist, Giacomo Scalari

**Affiliations:** 1 Quantum Optoelectronics Group, Institute of Quantum Electronics, ETH Zürich, 8093 Zürich, Switzerland; 2 Istituto di Fotonica e Nanotecnologie, CNR, Via del Fosso del Cavaliere 100, 00133 Rome, Italy

**Keywords:** Quantum cascade lasers, Integrated optics

Correction to: *Light: Science & Applications*

10.1038/s41377-022-01058-2 published online 24 December 2022

After the publication of this article^1^, we realized that a minor correction is required for Figure 5 (b, c) and the corresponding article text, due to a data analysis error in the reconstructed phase differences in the measured SWIFT spectrum.

**Figure 5 correction**

In the original article version, there was a minor error in Fig. 5. Specifically, panel (b) was displaying scattered phase differences, however, this was an artifact due to an error in the data analysis.
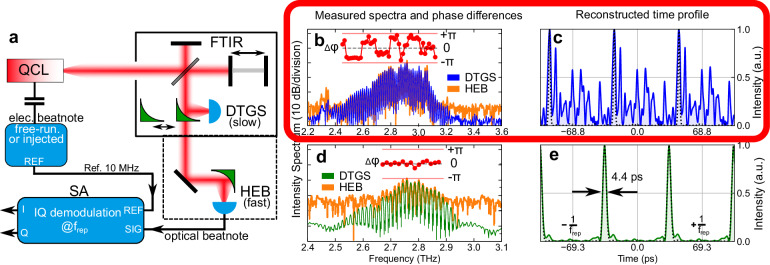


**Fig. 1** initially published version of Fig. 5, where panel **b** shows scattered phase differences and the reconstructed time profile in panel **c** displays an oscillatory behaviour.

After additional data analysis, the phase differences are not scattered but display a continuous (connected) relation. As shown in the corrected figure below, this also results in a modified reconstructed time profile, which now has a more pronounced amplitude-modulated periodic output intensity.
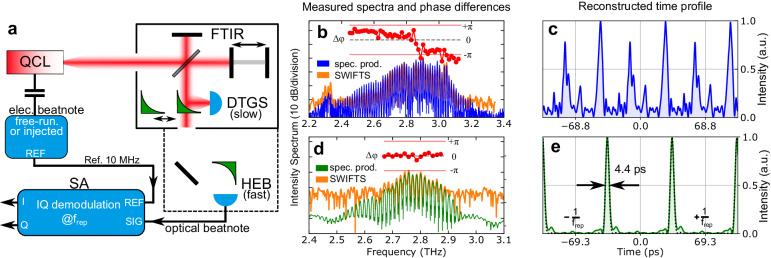


**Fig. 2** corrected figure with a continuous intermodal phase profile (panel **b**) and a reconstructed time profile with several pulses on top of a background intensity (panel **c**).

The updated figure caption for panels b, c is now (changes marked in **bold**):

**b, c** Measurements of a weakly-injected free-running ridge device show **relatively flat intermodal phase differences in two main groups, and the reconstructed time profile has a strongly amplitude-modulated periodic output intensity**.

The data analysis in panels (d, e) has been correct in the original publication and remains unchanged. Additionally, we changed the legend labels in panels (b) and (d) to “spec. prod.” and “SWIFTS”, as this is more consistent with other publications showing SWIFTS measurement results.

**Text correction**

Related to the modified Fig. 5, parts of the text are updated accordingly (changes marked in **bold**).

PDF page 7, top left:

… are shown in Fig. 5b, **where the spectrum product measured with a slow DTGS detector (blue) and the SWIFT spectrum measured with the HEB (orange)** have a good overlap and … The extracted **intermodal phase differences are flat over a large part of the spectrum but separated into several groups**. The reconstructed time profile produces a **periodic waveform with significant amplitude modulation, in particular, several pulses on top of a background intensity**, as shown in Fig. 5c.

PDF page 7, bottom left:

… as short as 4.4 ps. In this case, **the SWIFT spectrum** measurement with the HEB (orange) suffers from a worse signal-to-noise ratio as compared to **the spectrum product obtained with** the DTGS detector (green).

We would like to apologize for any inconvenience this may have caused.

The original publication has been corrected.
