# Supplementary material for: Planarized THz quantum cascade lasers for broadband coherent photonics
Source: Light Sci Appl. 2022 Dec 24;11:347. doi: 10.1038/s41377-022-01058-2 (PMC9789948; doi:10.1038/s41377-022-01058-2)
Supplement: Supplementary file 1 — Supplementary Material [file 41377_2022_1058_MOESM1_ESM.pdf]

Supplementary Information for
Planarized THz quantum cascade lasers for broadband coherent photonics

Urban Senica,[1] Andres Forrer,[1] Tudor Olariu,[1] Paolo Micheletti,[1] Sara Cibella,[2] Guido Torrioli,[2] Mattias Beck,[1] Jérôme Faist,[1] and Giacomo Scalari[1]

[1] *Quantum Optoelectronics Group, Institute of Quantum Electronics, ETH Zürich, 8093 Zürich, Switzerland*
[2] *Istituto di Fotonica e Nanotecnologie, CNR, Via del Fosso del Cavaliere 100, 00133 Rome, Italy*

I. THERMAL PROPERTIES

Here we elaborate in more detail the improved heat dissipation properties, due to the lateral heat transport through the extended top metallization and the BCB polymer. The general setup for the COMSOL 2D thermal simulations is illustrated in Fig. S1. In the computation, typical maximum operating bias conditions were used (11 V, 400 A cm^{-2}) for a heat sink temperature of 100 K, and the following material heat conductivites were considered: Cu = 320 W m^{-1}K^{-1}, active region = 5 W m^{-1}K^{-1} [S1], GaAs substrate = 100 W m^{-1}K^{-1}, BCB = 0.15 W m^{-1}K^{-1} (half of the listed conductivity at room temperature) [S2, S3]. Simulation results in Fig. S2(a) show that comparing a 40 µm wide planarized and a standard waveguide, the maximum temperature inside the active region is reduced by around 7 K.

To extract the experimental thermal properties of fabricated devices, we measured the threshold current density in pulsed and continuous wave (CW) operation as a function of increasing heat sink temperature [S4]. In pulsed mode, short pulses (150 ns) and low duty cycles (0.015%) were used to minimize sample heating above the set heat sink temperature. The results for 20 µm and 40 µm wide planarized ridge waveguides are presented in Fig. S2(b). The fitting function $J_{\mathrm{thr}} = J_0 \cdot \exp\left(\frac{T}{T_0}\right)$ has been applied using the last ∼5 high temperature points (solid lines in the graphs), with J_0 and T_0 as the fit parameters. The specific thermal conductance G was calculated using the expression

$$G = \frac{V_{\mathrm{thr}} \cdot J_{\mathrm{thr}}}{T_{\mathrm{p}} - T}, \tag{S1}$$

where CW values were used for V_{thr}, J_{thr}, and $T_{\mathrm{p}} = T_0 \cdot \ln\left(\frac{J_{\mathrm{thr}}}{J_0}\right)$ [S4]. The extracted thermal conductivity values are 250 W K^{-1}cm^{-2} at 60 K for the 20 µm wide waveguide and 130 W K^{-1}cm^{-2} at 90 K for the 40 µm wide waveguide. The latter value is especially interesting when compared to the data from our previous work [S5], with a computed thermal conductivity value of 80 W K^{-1}cm^{-2} at 90 K for a standard double metal waveguide with a width of 86 µm. Since the same active region and operating temperatures were used in the comparison, the use of a narrower planarized waveguide results in a factor of 1.6 higher thermal conductivity value for this specific case.

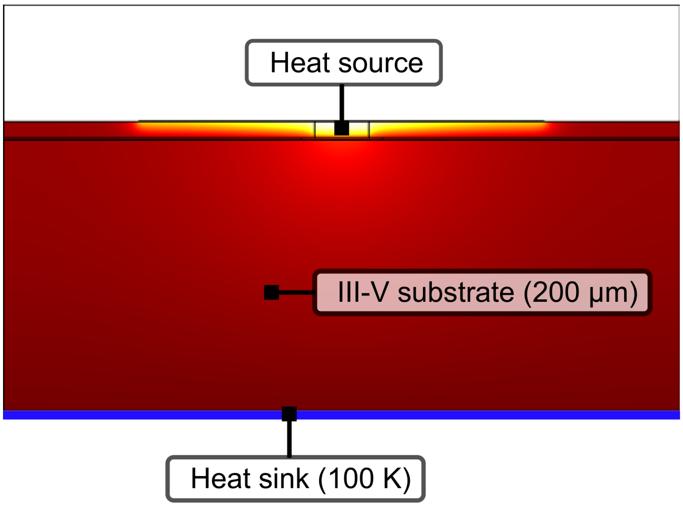

FIG. S1: General setup of the COMSOL 2D thermal simulation, where a heat source is positioned inside the cross section of the active waveguide, with an input power defined with the operating voltage and current of the device. A heat sink with a fixed temperature of 100 K is defined at the bottom of the GaAs substrate with a typical thickness of 200 µm. The material parameters are listed in the text.

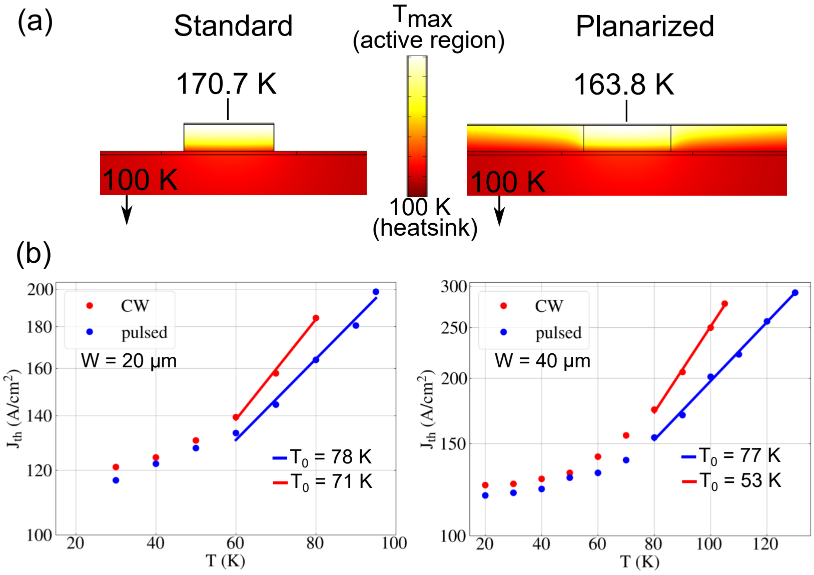

FIG. S2: **(a)** COMSOL 2D heat dissipation simulation for a 40 µm device at maximum operating conditions (V = 11 V, J = 400 A cm^{-2}) shows around 7 K lower maximum temperature in the active region for the planarized waveguide. The heat sink temperature is set to 100 K, and the material conductivities are Cu = 320 W $m^{-1}K^{-1}$, AR = 5 W $m^{-1}K^{-1}$, GaAs substrate = 100 W $m^{-1}K^{-1}$, BCB = 0.15 W $m^{-1}K^{-1}$. **(b)** Measured threshold current densities vs. heat sink temperature for 20 µm and 40 µm wide planarized waveguides, with the corresponding T_0 fit parameters.

In Fig. S3 we show 2D COMSOL simulation results for a varying active waveguide width, using the same parameters and settings as in Fig. S2(a). Comparing typical fabricated waveguide widths (80 µm for standard and 40 µm for planarized waveguides), the maximum temperatures inside the active region are 182.5 K and 164.0 K, respectively. This is a difference of nearly 20 K in favour of the planarized waveguide, at maximum laser bias conditions and at a heat sink temperature of 100 K.

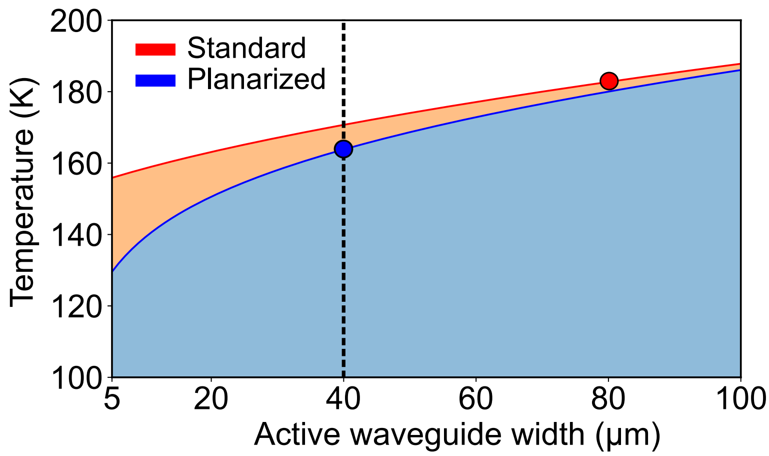

FIG. S3: Thermal simulation sweep of the waveguide width for a heat sink temperature of 100 K at the maximum laser bias condition. The dashed line is the comparison case highlighted in Fig. S2(a), while the circles mark typical fabricated active waveguide widths. The narrower waveguide widths and increased heat dissipation in planarized devices contribute to an improved, high temperature CW performance.

II. RF PROPERTIES

As shown in the main text in Fig. 3(b), the main origin of modified RF properties is the spreading of the field below the whole region with the extended top metallization. Here, we highlight results of a more detailed numerical simulation study.

The computed RF impedance for planarized waveguides stays almost constant for a varying active waveguide width with a fixed top contact width of 300 µm, as can be seen in Fig. 3(c) in the main text. Since the planarized waveguide impedance depends dominantly on the top contact width, it is then possible to decouple the design of RF and THz waveguide properties. We illustrate this idea in Fig. S4, where the computed impedance for a planarized waveguide with a fixed active waveguide width of 40 µm can be tuned between 4 Ω and 22 Ω by varying only the extended top contact width. This could be exploited by fabricating, for example, a corrugated shape of the extended metallization for custom RF properties, or also connecting to other in-plane coupling or surface-emitting RF antenna elements.

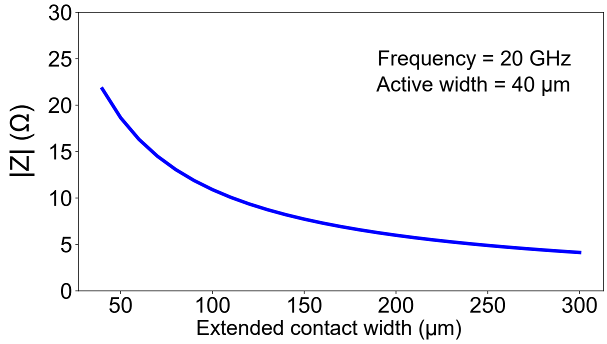

FIG. S4: 2D COMSOL impedance computation of a planarized waveguide with an active waveguide width of 40 µm and a varying extended top contact width at a frequency of 20 GHz.

We also performed 3D numerical simulations of the RF field of both standard and planarized waveguides. The simulation setup is illustrated in Fig. S5, where a TM source is defined inside of the active region waveguide, while the open boundary conditions allow to simulate and compute radiative losses for a variable source frequency.

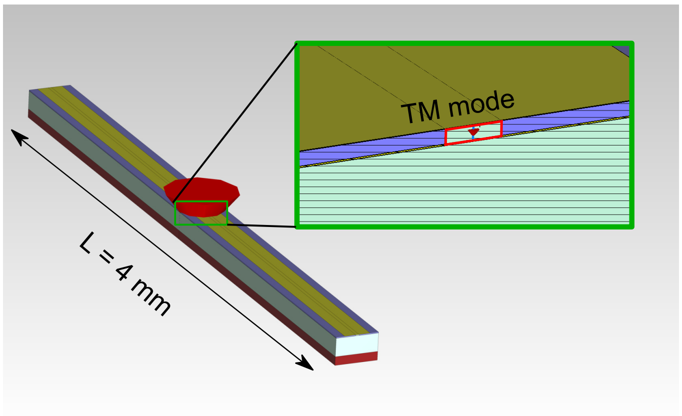

FIG. S5: 3D RF simulation setup, where a TM source is injected at the center of a 4 mm long waveguide, with open boundary conditions to simulate radiative losses.

In Fig. S6, we compare the computed reflectivities for a standard and a planarized waveguide with a length of 4 mm. To reflect typical fabricated devices, the planarized waveguide active waveguide width is 40 µm, while the standard waveguide is 80 µm wide. Here, a fixed refractive index of 3.6 has been used for the active region, while gold was modeled as a frequency-dependent lossy metal. For this waveguide length, the expected fundamental f_{rep} lies at 10 GHz. In the case of the standard waveguide (red curves), this is very close to the first microwave resonance. Due to the lower effective index, the first microwave resonance of the planarized waveguide is detuned to much higher frequencies, with a flat response close to f_{rep}. The latter waveguide also has lower radiative losses due to the lower

waveguide impedance, as can be seen for example by comparing the off-resonance reflectivities of both waveguides at around 30 GHz (0.977 vs 0.936 at 28.74 GHz). By extracting the FWHM width of the first resonance of the standard and planarized waveguide resonances (at 11.60 GHz and 19.45 GHz), we extract Q factors of 6.7 and 10.1 from the expression $Q = f_R/\Delta f$, where f_R is the position of the resonance and Δf the FWHM width.

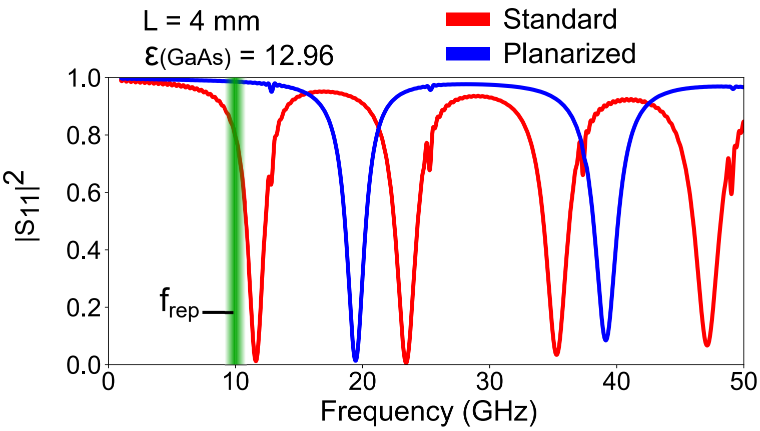

FIG. S6: 3D numerical simulation results of 4 mm long standard and planarized waveguides, with the computed reflectivities.

It was shown in Ref. [S6] that for standard double metal waveguides, the resonance of the fundamental microwave waveguide mode can coincide with the THz mode spacing f_{rep}. This is mainly due to a matching between the effective index of the microwave mode to the group index of the THz mode, i.e., $n_{\mathrm{eff}}^{\mathrm{GHz}} \simeq n_{\mathrm{g}}^{\mathrm{THz}}$. There, the authors modeled the active region with an effective bulk permittivity of $\varepsilon = 25$ to include the effect of the carriers (doping), and showed the resonance position matching occurs for a specific waveguide width. We simulated the planarized and standard waveguides also with this effective bulk permittivity, and the resulting reflectivities are in Fig. S7. It can be seen that the fundamental microwave resonance for the standard waveguide has now shifted below f_{rep} (11.6 GHz to 8.4 GHz), while that of the planarized waveguide microwave resonances is still detuned to much higher frequencies (19.4 GHz to 16.3 GHz). The relative frequency shifts are -28% and -16% for the standard and planarized waveguide, respectively. This supports the observation that the RF properties of planarized waveguides depend less on the active region waveguide properties.

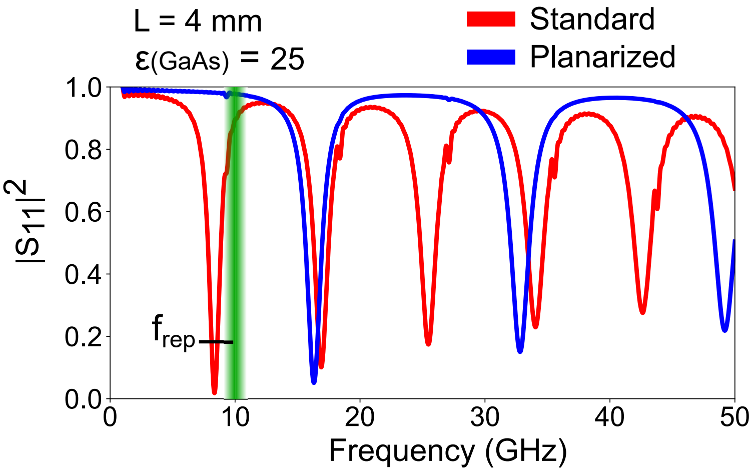

FIG. S7: 3D numerical simulation results of 4 mm long standard and planarized waveguides, with the computed reflectivities for an increased active region premittivity of ε(GaAs)=25

III. RF BEATNOTE MAPS

Here we plot the RF beatnote maps of several devices from the main text, namely the ridge device in Fig. S8 and two active-passive devices in Fig. S9 and Fig. S10. The bias voltage is swept while recording the RF spectrum with a spectrum analyzer. The blue boxes highlight the bias range with a single RF beatnote, a signature of comb operation.

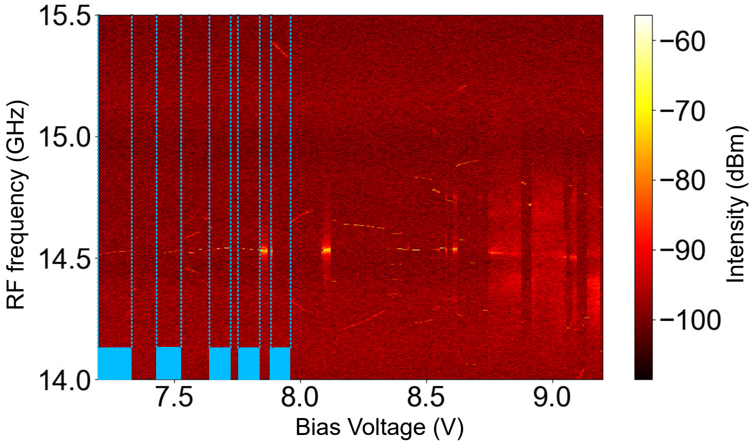

FIG. S8: Beatnote map, ridge device from the main text, Fig. 3. Solid blue boxes with lines mark the region with a single RF beatnote, a signature of comb operation.

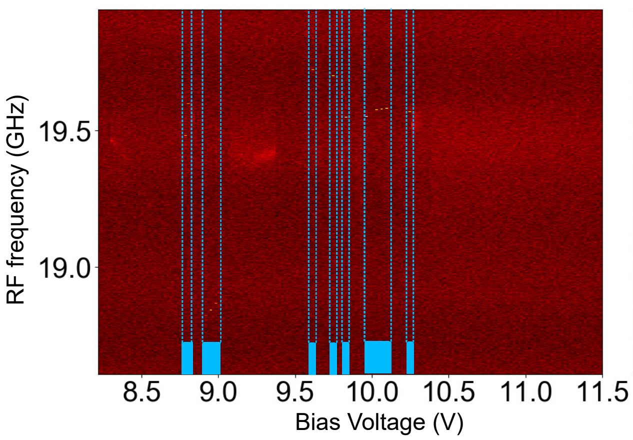

FIG. S9: Beatnote map, active-passive device from the main text, Fig. 5.

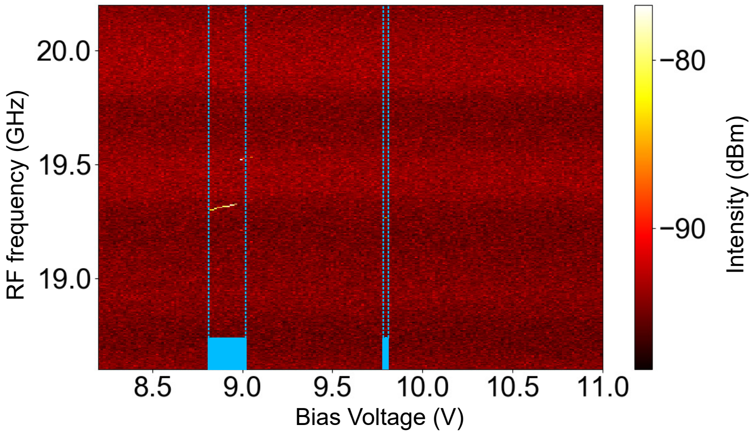

FIG. S10: Beatnote map of a second active-passive device, not shown in the main text.

References

[S1] Scamarcio, G. *et al.* Nanoscale heat transfer in quantum cascade lasers. *Physica E: Low-Dimensional Systems and Nanostructures* **40**, 1780–1784 (2008).
[S2] Choy, C. L. Thermal conductivity of polymers. *Polymer* **18**, 984–1004 (1977).
[S3] Greig, D. Low temperature thermal conductivity of polymers. *Cryogenics* **28**, 243–247 (1988).
[S4] Faist, J. *Quantum cascade lasers* (Oxford University Press, Oxford, United Kingdom, 2013), first edition edn.
[S5] Forrer, A., Bosco, L., Beck, M., Faist, J. & Scalari, G. RF Injection of THz QCL Combs at 80 K Emitting over 700 GHz Spectral Bandwidth. *Photonics* **7**, 9 (2020).
[S6] Maineult, W. *et al.* Microwave modulation of terahertz quantum cascade lasers: a transmission-line approach. *Applied Physics Letters* **96**, 021108 (2010).
